# Vitamin D Deficiency in Institutionalized Older Adults: Associations with Supplementation Practices but Not with Cognitive Decline or Dementia

**DOI:** 10.3390/nu18071078

**Published:** 2026-03-27

**Authors:** Larissa David Soares, Myrella Teixeira Rosales, Bruna Costa Silveira, Alice Moreira Rizzolli, Caroline Helen Santos Gonçalves Mazala, Isabela Thurow Lemes, Fabiana Da Silveira Santos Sinnott, Thiago Falson Santana, Érica Paiva Espinosa, Eduarda Neutzling Drawanz, Ana Beatriz Gonçalves Araújo, Nathalia Passos Moura, Aline Longoni, Diogo Onofre Souza, Maria Noel Marzano Rodrigues, Adriano Martimbianco De Assis

**Affiliations:** 1 Graduate Program in Health and Behavior, Center of Health Sciences, Laboratory of Clinical Neurosciences, Catholic University of Pelotas (UCPel), Pelotas 96015-560, RS, Brazil; larissa.soares@sou.ucpel.edu.br (L.D.S.); alice.rizzolli@sou.ucpel.edu.br (A.M.R.); caroline-helen@hotmail.com (C.H.S.G.M.);; 2 Graduate Program in Biological Sciences: Biochemistry, Institute of Basic Health Sciences, Federal University of Rio Grande Do Sul (UFRGS), Porto Alegre 90035-003, RS, Brazil; isabelalemesfisioterapeuta@gmail.com (I.T.L.);

**Keywords:** dementia, vitamin D, institutionalized older adults, cognitive decline, institutionalization, hypovitaminosis D

## Abstract

Background/Objectives: Population aging has been accompanied by increased institutionalization of older adults and a high prevalence of vitamin D deficiency in this group. Although the literature suggests a possible relationship between vitamin D and cognition, findings remain inconsistent, particularly in institutional settings. This cross-sectional study aimed to investigate factors associated with vitamin D deficiency in institutionalized older adults, emphasizing the role of vitamin D supplementation and length of institutionalization, as well as to evaluate the association between serum vitamin D levels, cognitive decline, and dementia. Methods: A total of 104 older adults living in different long-term care institutions (LTCFs) in the city of Pelotas, RS, Brazil, were evaluated. Sociodemographic, clinical, and nutritional data were collected via interviews and medical record review. Serum 25-hydroxyvitamin D levels were categorized according to the Institute of Medicine cutoffs (<20 ng/mL and ≥20 ng/mL). Cognitive decline was assessed using the Mini-Mental State Examination, and dementia was evaluated with the Clinical Dementia Rating scale. Analyses included bivariate tests and binary logistic regression. Results: A high prevalence of vitamin D deficiency (52.9%), cognitive decline (83.6%), and questionable or mild dementia (79.4%) was observed. In multivariate analysis, vitamin D supplementation remained independently associated with vitamin D deficiency, whereas no significant association was observed between vitamin D levels and cognitive decline or dementia. Conclusions: Vitamin D deficiency in institutionalized older adults is predominantly associated with contextual and care-related factors rather than cognitive impairment, highlighting the importance of systematic nutritional monitoring and vitamin D supplementation strategies in institutional settings.

## 1. Introduction

Population aging has emerged as a rapidly progressing global phenomenon, consolidating itself as a major contemporary medical and social challenge [1]. This demographic transition has been profound. While the proportion of older adults was relatively small in 1950, projections for 2050 indicate that individuals aged 60 years and older will, for the first time, outnumber adolescents, potentially accounting for up to 38% of a country’s population [1,2].

Aging is a heterogeneous and multidimensional process encompassing biological, psychological, and social changes [3]. Although universal, its manifestations are highly individual and depend on complex interactions between genetic background, environmental exposures, and lifestyle factors. From a biological perspective, aging is characterized by the progressive decline of cellular repair mechanisms and the loss of homeostatic regulation [4]. These processes contribute to functional deterioration and increase susceptibility to chronic conditions such as diabetes; cardiovascular diseases; hypertension; and neurodegenerative disorders, including dementia [5,6].

Aging represents the main risk factor for age-related dementia (ARD). The prevalence of ARD rises markedly after the age of 65, nearly doubling every five years [7,8]. By the ninth decade of life (80–89 years), approximately one in three individuals may meet diagnostic criteria for dementia or present significant cognitive impairment [8,9]. Even in the absence of a formal dementia diagnosis, aging itself is associated with measurable cognitive decline and neurodegenerative changes. Basic cognitive domains, including processing speed, reasoning, episodic memory, and visuospatial abilities, tend to deteriorate progressively during later life [8,10].

Parallel to population aging, an increase in institutionalization has been observed worldwide, resulting in a growing number of older adults residing in long-term care facilities (LTCFs) [11]. Limited functional capacity and insufficient support for activities of daily living are among the main determinants of this transition [12]. Although institutionalization may be necessary in specific circumstances, residence in LTCFs is often associated with reduced autonomy, lower perceived quality of life, and potential acceleration of functional and cognitive decline, highlighting the importance of adequate health and nutritional care in these settings [13].

Older adults living in institutional environments are particularly vulnerable to nutritional deficiencies, with vitamin D deficiency representing one of the most prevalent and clinically relevant conditions in this population. Insufficient vitamin D levels negatively affect quality of life by increasing the risk of frailty, impairing muscle function and mobility, and potentially influencing cognitive health [14]. Aging is accompanied by a reduced capacity of the skin to synthesize vitamin D, in addition to lower sun exposure, especially among institutionalized individuals [15]. Prevention of hypovitaminosis D relies on adequate sunlight exposure, dietary intake of fortified foods and natural sources such as dairy products and fish, and, when necessary, vitamin D supplementation [16].

Vitamin D is an essential fat-soluble micronutrient that exists in two biologically inactive isoforms: ergocalciferol (vitamin D_2_) and cholecalciferol (vitamin D_3_) [17]. Vitamin D_2_ is obtained mainly from plant-based sources and supplementation, whereas vitamin D_3_ can be acquired through diet or synthesized endogenously in the skin following exposure to ultraviolet B (UVB) radiation [18]. Beyond its classical role in calcium and bone metabolism, vitamin D has been implicated in several biological processes related to aging, including mitochondrial quality control, oxidative stress modulation, inflammation, and DNA damage repair [19].

Vitamin D has also been implicated in several neurobiological processes that may influence cognitive function. Experimental and clinical studies suggest that vitamin D participates in neuroprotection through the modulation of neuroinflammatory pathways, the regulation of neuronal calcium homeostasis, antioxidant activity, and potential effects on amyloid metabolism [20,21]. Observational studies have reported associations between low serum 25(OH)D concentrations and increased risk of cognitive decline and dementia [22,23,24]. However, findings remain heterogeneous, particularly in institutionalized older populations where multiple comorbidities and environmental factors may influence both vitamin D status and cognitive health [25,26].

Given the age-related decline in endogenous vitamin D synthesis, older adults—particularly those living in LTCFs—may experience a vicious cycle in which aging promotes vitamin D deficiency, which, in turn, may exacerbate age-related functional decline [27]. Vitamin D deficiency is highly prevalent worldwide and constitutes a major public health concern, with reported prevalence rates exceeding 90% in certain populations, including in Brazil [28]. However, despite growing interest in the potential relationship between vitamin D status and cognitive outcomes, evidence remains inconsistent, especially in institutionalized older adults [26].

In this context, this study aimed to investigate factors associated with vitamin D deficiency in institutionalized older adults, with particular emphasis on vitamin D supplementation and length of institutionalization, as well as to evaluate the association between serum vitamin D levels, cognitive decline, and dementia among residents of long-term care institutions in Pelotas, Rio Grande do Sul, Brazil.

## 2. Materials and Methods

### 2.1. Study Design

This study was part of a larger population-based investigation that evaluated institutionalized and non-institutionalized older adults living in the urban area of Pelotas, Rio Grande do Sul, Brazil. The present analysis corresponds to a cross-sectional study, in which participants were selected from the institutionalized subsample, based on census data provided by the Brazilian Institute of Geography and Statistics [29]. Data collection was carried out between June and September 2025.

### 2.2. Study Population and Sample

The municipality of Pelotas is divided into seven administrative regions—Areal, Barragem, Centro, Fragata, Laranjal/Z3, São Gonçalo, and Três Vendas—which differ in the demographic density of older adults. The total distribution of long-term care institutions for older adults (LTCFs) in the urban area is as follows: Areal (4), Centro (42), Fragata (7), Laranjal/Z3 (2), Três Vendas (6), São Gonçalo (1), and Barragem (0). Among these, the LTCFs included in this study were distributed as follows: Areal (3), Centro (4), Fragata (2), Três Vendas (3), and São Gonçalo (1), with no participating institutions in Laranjal/Z3 or Barragem.

A total of 26 LTCFs were contacted, of which 13 agreed to participate. All institutions were contacted to present the research project and invite participation. In those that agreed, the institutional manager signed a formal Institutional Agreement Term, authorizing this study to be conducted. Data collection schedules were then defined according to each institution’s routine, respecting their preferred days and times for receiving the research team.

The 104 participants were recruited from 13 long-term care facilities (LTCFs) distributed across five geographic regions of the city to ensure a diverse representation of the institutionalized population. Participating facilities were identified using alphanumeric codes (LTCF1–LTCF13) throughout the manuscript rather than by their institutional names to preserve institutional confidentiality. In each facility, a census-based recruitment was performed, inviting all residents who met the inclusion criteria. The distribution of participants per region and facility was as follows: Três Vendas (LTCF 1, *n* = 14; LTCF 2, *n* = 12; LTCF 3, *n* = 11); Areal (LTCF 4, *n* = 7; LTCF 5, *n* = 4; LTCF 6, *n* = 5); Fragata (LTCF 7, *n* = 4; LTCF 8, *n* = 5); Centro (LTCF 9, *n* = 14; LTCF 10, *n* = 8; LTCF 11, *n* = 8; LTCF 12, *n* = 4); and São Gonçalo (LTCF 13, *n* = 8). The number of residents per facility ranged from 4 to 14, with no single institution representing more than 13.5% of the total study population.

### 2.3. Ethical Considerations

This study was approved by the Research Ethics Committee of the Catholic University of Pelotas (approval number 7.448.639; Certificate of Presentation for Ethical Appraisal—CAAE No. 86772725.1.0000.5339) on 2 April 2025. On the scheduled data collection days, older adults were approached by trained interviewers and invited to participate. Participation was voluntary, and it was emphasized that individual consent was required regardless of institutional authorization. All procedures and instruments were explained in detail. Written informed consent was obtained from all participants or from their legal representatives by signing an Informed Consent Form.

### 2.4. Inclusion and Exclusion Criteria

Eligible participants were institutionalized older adults of both sexes, of any race/ethnicity, aged between 60 and 95 years, residing in LTCFs located in the urban area of Pelotas. Individuals with severe cognitive impairment who did not have a legal representative or an appropriate informant were excluded.

### 2.5. Procedures and Instruments

#### 2.5.1. Sample Characterization

A structured questionnaire was used to characterize the sample and included identification data (initials, date of birth, sex, race/ethnicity, and educational level); personal information (length of institutionalization and physical activity); and clinical data (use of medications and presence of comorbidities), such as stroke, anemia, asthma, diabetes mellitus, coronary artery disease, chronic obstructive pulmonary disease, epilepsy, hypertension, heart failure, gastrointestinal ulcer, mild cognitive impairment, and dementia.

#### 2.5.2. Vidsun Questionnaire

The VIDSUN questionnaire is an instrument designed to classify the risk of vitamin D insufficiency. It assesses multiple risk factors, including sun exposure (frequency, sunscreen use, average exposure time in the previous week, and use of tanning booths), dietary habits (consumption of milk, cod liver oil, or vitamin D-rich fish), supplement use (multivitamins and specific vitamin D supplements, including doses), and the presence of frequent muscle pain. Vitamin D supplementation was further categorized according to weekly dose (≤7000 IU/week vs. >7000 IU/week) to explore potential dose–response patterns. Additionally, self-identified race/ethnicity, body mass index (BMI)—calculated from measured weight and height—and Fitzpatrick skin phototype were collected. These variables generate a composite score, in which a final score ≤2.25 indicates a higher probability of vitamin D insufficiency [30]. In our study, the VIDSUN score was utilized as an exploratory screening tool to evaluate its predictive value against direct biochemical measurement in an institutionalized population.

#### 2.5.3. Cognitive Assessment

Cognitive screening was performed using the Mini-Mental State Examination (MMSE). This instrument evaluates five main cognitive domains: orientation (temporal and spatial), memory (immediate and delayed recall), attention and calculation, and language (including naming, repetition, reading, writing, and copying). Education-adjusted cutoff points were applied to determine the presence or absence of cognitive decline [31].

#### 2.5.4. Dementia Assessment

Dementia staging was conducted using the Clinical Dementia Rating (CDR) scale. The CDR evaluates six cognitive and functional domains: memory, orientation, judgment and problem solving, community affairs, home and hobbies, and personal care. Each domain is scored from 0 (no impairment) to 3 (severe impairment). The global CDR score was calculated according to the standard algorithm, in which the memory domain is considered the primary determinant of overall dementia severity (0, 0.5, 1, 2, or 3) [32].

#### 2.5.5. Blood Collection and Biochemical Analysis

For the determination of serum vitamin D levels, blood samples were collected by a trained nurse via venipuncture and processed within 4 h. Samples were centrifuged at 3500 rpm for 15 min at 21 °C, and serum aliquots were stored at −80 °C for subsequent quantification of 25-hydroxyvitamin D. Serum 25-hydroxyvitamin D concentrations were measured using an automated electrochemiluminescence immunoassay (Atellica IM, Siemens, Munich, Germany) in a partner laboratory [33]. The results were expressed in ng/mL. Vitamin D deficiency was defined as serum 25-hydroxyvitamin D [25(OH)D] concentration <20 ng/mL, according to the Institute of Medicine (IOM) recommendations, which are widely used in epidemiological studies [33] and clinical practice focusing on bone health outcomes.

### 2.6. Statistical Analysis

The data were collected using Google Forms and organized in Microsoft Excel spreadsheets. Statistical analyses were performed using IBM SPSS Statistics, version 27. Bivariate analyses were conducted using the chi-square test and Student’s *t*-test. Multivariate analysis was performed using binary logistic regression (enter method), including potential confounders identified in the literature and variables presenting *p*-values < 0.20 in the bivariate analyses. Associations were considered statistically significant at *p* ≤ 0.05. The regression models were used to evaluate the extent to which serum vitamin D levels could be predicted by independent participant characteristics, including sociodemographic variables (age), anthropometric measures (BMI), health-related factors (vitamin D supplementation), and contextual variables (length of institutionalization). For categorical variables, the first coded category was used as the reference. Regarding missing data, only one observation had missing information for the vitamin D supplementation variable. This observation was, therefore, excluded from the analyses involving this variable, and a complete-case approach was applied [34].

## 3. Results

Table 1 presents the sociodemographic, clinical, anthropometric, and contextual characteristics of the institutionalized older adults included in this study, as well as their associations with vitamin D deficiency. The sample comprised 104 participants, with a mean age of 77.15 years (SD = 8.2). A predominance of women (68.3%) and individuals with self-reported white skin color (84.6%) was observed. Most participants (71.2%) had low educational attainment, defined as up to seven years of formal education. Regarding clinical characteristics, most participants were classified as having questionable or mild dementia (79.8%), and polypharmacy was common, with 54.8% of older adults using between five and eight medications. Most participants (81.7%) presented up to three comorbid conditions. The prevalence of vitamin D deficiency, cognitive decline, and questionable or mild dementia in the study population was 52.9%, 83.6%, and 79.8%, respectively.

The analysis of the association according to weekly supplementation dose was statistically significant (Table 1; *p* < 0.001). Among individuals who did not receive supplementation (*n* = 82), the prevalence of deficiency was 67.1%, while only 32.9% had sufficient vitamin D levels. In contrast, among participants receiving ≤7000 IU/week (*n* = 17) and those receiving >7000 IU/week (*n* = 5), 100% of individuals did not present vitamin D deficiency. No cases of deficiency were recorded among participants using any supplementation dose, highlighting the determinant role of supplementation in maintaining adequate vitamin D status in this population.

No statistically significant associations (*p* > 0.05) were observed between serum vitamin D levels and sociodemographic variables, including age (*p* = 0.061), sex (*p* = 0.377), educational level (*p* = 0.808), and skin color (*p* = 0.900). Similarly, no significant differences were found between participants with and without vitamin D deficiency regarding clinical and anthropometric variables, such as dementia stage (CDR) (*p* = 0.941), medication use (*p* = 0.380), number of comorbidities (*p* = 0.833), body mass index (BMI) (*p* = 0.287), presence of cognitive decline assessed by the MMSE (*p* = 0.370), or probability of vitamin D deficiency according to the VIDSUN questionnaire (*p* = 0.183). Additional analyses exploring the relationship between vitamin D status and cognitive outcomes, including MMSE as a continuous variable and stratified CDR categories, are presented in Table 2.

The mean length of institutionalization was shorter among older adults with vitamin D deficiency (22.72 months) compared with those with adequate vitamin D levels (29.63 months), although this difference did not reach statistical significance in the bivariate analysis (*p* = 0.085). Although not statistically significant, older adults with vitamin D deficiency were more frequently female (71.9%), had low educational attainment (≤7 years of schooling; 70.2%), and were predominantly of white skin color.

For the multivariate analysis (Table 3), binary logistic regression (enter method) was performed considering potential confounding factors described in the literature. Associations were considered statistically significant when *p* ≤ 0.05.

The model included variables related to participant characteristics, including sociodemographic (age), anthropometric (BMI), health-related (weekly vitamin D supplementation dose), and contextual (length of institutionalization) factors. For categorical variables, the first level in the coding scheme was used as the reference category. The model was statistically significant [χ^2^(5) = 23.560 and *p* < 0.001; Nagelkerke R^2^ = 0.326] and correctly classified 67.9% of the cases (53.7% among participants without vitamin D deficiency and 81.4% among those with vitamin D deficiency).

In the multivariable model, neither age (OR = 0.964; 95% CI: 0.904–1.027; *p* = 0.252) nor BMI (OR = 1.078; 95% CI: 0.884–1.314; *p* = 0.460) was significantly associated with vitamin D deficiency. In contrast, the weekly vitamin D supplementation dose showed a significant overall association with the outcome (*p* = 0.004). Compared with participants who did not receive supplementation, those receiving ≤7000 IU per week had significantly different odds of vitamin D deficiency (OR = 27.95; 95% CI: 3.247–240.659; *p* = 0.002). For individuals receiving >7000 IU per week, an association in the same direction was observed, although it did not reach statistical significance (OR = 6.65; 95% CI: 0.622–71.009; *p* = 0.117).

## 4. Discussion

The main findings of this study indicate that vitamin D deficiency in institutionalized older adults is predominantly associated with contextual and care-related factors, particularly the absence of supplementation, rather than with cognitive decline or the presence of dementia. The observed association between vitamin D supplementation and deficiency likely reflects reverse causality, as supplementation in long-term care settings is frequently initiated after deficiency is detected. In this context, supplementation may act as a marker of previous diagnosis rather than as a protective factor against deficiency.

One of the most relevant findings of the present study is the high prevalence of vitamin D deficiency among institutionalized older adults (52.9%) with serum concentrations of ≤20 ng/mL. This prevalence is consistent with previous studies reporting high rates of hypovitaminosis D in older adults living in long-term care institutions. This high prevalence is consistent with studies in long-term care settings, where limited sun exposure and dietary intake are common. The situation is even more concerning when considering the threshold for optimal bone health (>30 ng/mL), as 84.6% of the participants exhibited inadequate vitamin D levels, with a mean concentration of 21.12 ± 10.14 ng/mL. These results underscore the systemic nature of vitamin D inadequacy in LTCFs across different regions of the city, regardless of the specific facility’s size or location. Moreover, different organizations propose alternative thresholds for defining vitamin D deficiency, such as <30 ng/mL, as suggested by the Endocrine Society. These differences in classification criteria may influence the estimated prevalence of deficiency across studies. In this study, we used the Institute of Medicine cutoff (<20 ng/mL), a conservative and widely used reference in population-based research [33]. Serum vitamin D levels and ultraviolet (UV) exposure indices are consistently lower in institutionalized older adults compared with those living in the community [35,36]. Hypovitaminosis D is, therefore, highly prevalent in older populations and is particularly pronounced among individuals with chronic comorbidities, polypharmacy, and limited sun exposure, conditions commonly observed in long-term care settings [16,37,38]. As a result, bedridden and institutionalized older adults constitute a high-risk group for multiple nutritional deficiencies, including vitamin D [39].

The severity of vitamin D deficiency observed in our study warrants particular attention, as the lowest measured serum concentration was 2.8 ng/mL. Such low levels may result from insufficient dietary intake of vitamin D-rich foods, such as milk, cheese, egg yolk, and fatty fish (e.g., salmon, tuna, and mackerel), combined with limited sun exposure [40]. Although cutaneous synthesis is recognized as the primary mechanism for maintaining adequate vitamin D levels, inadequate dietary intake further exacerbates the risk of deficiency [41]. In long-term care institutions, diets often lack specific optimization for vitamin D due to reduced appetite, physical frailty, or dietary restrictions among residents. Socioeconomic factors also play a role, as fish and other vitamin D-rich foods are often more expensive, further increasing the risk of deficiency and compromising the health of institutionalized older adults [42,43].

The institutional environment frequently restricts exposure to sunlight, the main determinant of vitamin D synthesis, helping to explain another key finding of this study: longer length of institutionalization emerged as an independent predictor of vitamin D deficiency. This result highlights a direct and progressive relationship between duration of institutional stay and lower serum vitamin D levels, with each additional month of institutionalization increasing the likelihood of deficiency [44]. Regarding the clinical screening of vitamin D status, the VIDSUN questionnaire did not show a significant association with serum 25(OH)D levels in our final model. This lack of predictive power can be attributed to the high homogeneity and the high prevalence of vitamin D inadequacy (84.6% < 30 ng/mL) in this population. In institutionalized settings, lifestyle factors such as sun exposure and diet are often standardized by the facility’s routine, which reduces the variability in the questionnaire’s components. Consequently, our findings suggest that while such tools may be useful in community-dwelling populations, they may have limited sensitivity for discriminating vitamin D status in long-term care facilities, where biochemical assessment remains the essential gold standard for diagnosis.

Low vitamin D levels have been associated with an increased risk of dementia and worsening cognitive decline, supported by evidence suggesting that vitamin D acts as an important neurosteroid in the aging process [45]. Giustina et al. [41] reported significant associations between vitamin D status and performance in the Mini-Mental State Examination, independent of the presence of cognitive impairment. However, in this study, no significant association was observed between serum vitamin D levels and dementia stage (CDR) or cognitive decline assessed by the MMSE. Additional exploratory analyses using MMSE as a continuous variable and stratified CDR categories also did not demonstrate significant associations with vitamin D status (Table 2). This finding is consistent with recent observational research that also reported no significant relationship between 25(OH)D levels and cognitive test scores after adjustment for confounders [46].

Several factors may explain the absence of association between vitamin D status and cognitive outcomes in this study. First, vitamin D levels among institutionalized older adults tend to be uniformly low, regardless of cognitive status [37]. This homogeneity may reduce variability and limit the ability to detect differences between groups. The high prevalence of cognitive impairment observed in the present sample is consistent with the clinical profile commonly reported in long-term care institutions. More than 80% of participants presented cognitive impairment, which substantially reduced variability in cognitive outcomes and may have limited the statistical power to detect potential associations between vitamin D status and cognitive measures. Second, the relationship between vitamin D and dementia is not always linear and may not be sufficiently robust to be detected in cross-sectional analyses [47]. Furthermore, this study identified a high prevalence of cognitive decline, questionable dementia, and mild dementia. Evidence suggests that the potential protective effects of vitamin D may be more evident before or during the earliest stages of cognitive decline, diminishing as the disease progresses and other pathophysiological mechanisms become dominant [23,47,48]. Many studies assess vitamin D levels before dementia diagnosis, indicating that its potential protective role may occur primarily during asymptomatic or early stages [23,47].

Among older adults who reported vitamin D supplementation (*n* = 22), only three presented vitamin D deficiency. Accordingly, supplementation emerged as an important independent predictor, with non-supplemented individuals presenting approximately ten times higher odds of deficiency. This finding reinforces the effectiveness of vitamin D supplementation as a strategy for correcting and maintaining adequate serum levels, particularly in institutional settings where sun exposure and dietary sources are limited [35]. Previous studies have also shown that institutionalized older adults rely more heavily on supplementation than on cutaneous synthesis to achieve adequate vitamin D levels due to reduced autonomy, limited outdoor time, and structural barriers within institutions. Current guidelines generally recommend year-round vitamin D supplementation for older adults [41,49]. Recent evidence also highlights the utility of blood-based biochemical parameters for identifying nutritional risk in older adults, underscoring the importance of systematic nutritional monitoring in institutionalized populations [50].

Some methodological considerations should be considered when interpreting these findings. The cross-sectional design precludes causal inference, and the association observed between vitamin D supplementation and deficiency may partly reflect reverse causality, as residents identified with low vitamin D levels may have been more likely to receive supplementation. Additionally, the high prevalence of cognitive impairment in the study population (over 80%) may have reduced variability in cognitive outcomes and limited the statistical power to detect potential associations between vitamin D status and cognitive decline. Serum 25(OH)D concentrations were measured using an immunoassay method commonly employed in clinical laboratories. Although widely used in epidemiological studies, immunoassays may show variability compared with reference methods such as liquid chromatography–tandem mass spectrometry (LC–MS/MS). Another limitation concerns institutional participation: of the 26 facilities contacted, 13 agreed to participate, which may have introduced institutional-level selection bias. Comorbidities were summarized as a numerical count to preserve model stability given the study sample size, limiting the evaluation of specific clinical conditions. Finally, because this study was conducted in long-term care institutions from a single municipality, caution is warranted when generalizing the findings to other settings. Nevertheless, this study provides relevant insight into institutional and care-related determinants of vitamin D deficiency among older adults living in long-term care settings.

## 5. Conclusions

In this sample of institutionalized older adults, vitamin D deficiency was highly prevalent and was associated with supplementation practices, likely reflecting clinical management following the detection of deficiency rather than a preventive strategy. No association was observed between vitamin D status and cognitive decline or dementia. However, the cross-sectional design and the limited variability in cognitive outcomes in this population may have reduced the ability to detect potential relationships between vitamin D status and cognitive measures. Taken together, these findings suggest that hypovitaminosis D in long-term care settings may be more closely related to institutional and care-related factors than to cognitive impairment, highlighting the importance of routine vitamin D monitoring and appropriate supplementation strategies in long-term care facilities.

## Figures and Tables

**Table 1:** Sociodemographic, clinical, anthropometric, and institutional characteristics of institutionalized older adults in Pelotas, southern Brazil, and their association with vitamin D deficiency (*n* = 104). Data are presented as absolute numbers (*n*) and percentages (%). A *p*-value of < 0.05 was considered statistically significant.

Variable	Total	Vitamin D Deficiency (Yes) *	Vitamin D Deficiency (No) *	*p*-Value
	*n* (%)	*n* (%)	*n* (%)	
Age, years, mean (SD) ^b^	77.15 (8.2)	77.60 (8.3)	76.62 (8.0)	0.547
Education level ^a^				0.808
Up to 7 years of schooling	74 (71.2)	40 (54.1)	34 (45.9)	
8 years of schooling or more	30 (28.8)	17 (56.6)	13 (43.4)	
Sex ^a^				0.377
Female	71 (68.3)	41 (57.7)	30 (42.3)	
Male	33 (31.7)	16 (48.5)	17 (51.5)	
Vitamin supplementation *^a^				<0.01
Yes	22 (21.4)	3 (13.6)	19 (86.4)	
No	81 (78.6)	53 (65.4)	28 (34.6)	
Vitamin D supplementation (weekly dose) ^d^				<0.01
No supplementation	82 (78.8)	55 (67.1)	27 (32.9)	
≤7000 IU/week	17 (16.3)	1 (5.9)	16 (94.1)	
>7000 IU/week	5 (4.8)	1 (20.0)	4 (80.0)	
Skin color ^a^				0.900
White	88 (84.6)	48 (54.5)	40 (45.5)	
Non-white	16 (15.7)	9 (56.2)	7 (43.8)	
CDR ^c^				0.941
Without dementia	11 (10.6)	6 (54.5)	5 (45.5)	
Questionable or mild dementia	83 (79.8)	45 (54.2)	38 (45.8)	
Moderate or severe dementia	10 (9.6)	6 (60)	4 (40)	
Medication use ^a^				0.380
Up to 4 medications	31 (29.8)	20 (64.5)	11 (35.5)	
5–8 medications	57 (54.8)	28 (49.1)	29 (50.9)	
9 or more medications	16 (15.4)	9 (56.2)	7 (43.8)	
Comorbidity ^a^				0.833
Up to 3 comorbidities	85 (81.7)	47 (55.3)	38 (44.7)	
>3 comorbidities	19 (18.3)	10 (52.6)	9 (47.4)	
Mini-Mental ^a^				0.370
With cognitive decline	87 (83.7)	46 (52.9)	41 (47.1)	
Without cognitive decline	17 (16.3)	11 (64.7)	6 (35.3)	
VIDSUN ^a^				0.183
Yes	24 (23.1)	16 (66.7)	8 (33.3)	
No	80 (76.9)	41 (51.2)	39 (48.8)	
Institutionalization time (in months), mean (SD) ^b^	25.9 (18.8)	22.72 (17.1)	29.63 (20.3)	0.085
BMI (body mass index), mean (SD) ^b^	25.7 (2.5)	25.56 (2.40)	26.12 (2.59)	0.287

* Variable with missing data. ^a^ Pearson’s chi-square test; distribution in the sample expressed as % (*n*). ^b^ Test t; distribution in the sample expressed as mean (DP). ^c^ Fischer test. ^d^ Maximum likelihood ratio chi-square test.

**Table 2:** Additional analyses exploring the association between vitamin D status and cognitive outcomes.

Variable	Total	Vitamin D Deficiency (Yes)	Vitamin D Deficiency (No)	*p*-Value
	*n* (%)	*n* (%)	*n* (%)	
Clinical Dementia Rating (CDR) ^a^				0.985
Without dementia (CDR = 0)	11 (10.6)	6 (54.5)	5 (45.5)	
Any level of dementia (CDR ≥ 0.5)	93 (89.4)	51 (54.8)	42 (45.2)	
MMSE score, mean (SD) ^b^	18.76 (5.7)	18.79 (6.0)	18.72 (5.3)	0.954

^a^ Fisher’s exact test; ^b^ Student’s *t*-test. MMSE: Mini-Mental State Examination. Values expressed as means (standard deviation).

**Table 3:** Multivariable logistic regression analysis identifying independent predictors of vitamin D deficiency among institutionalized older adults in Pelotas, southern Brazil (*n* = 104). The model was adjusted using the enter method. Odds ratios (ORs) with 95% confidence intervals (95% CIs) are presented.

Variable	OR	95% CI	*p*-Value
Age (years)	0.964	0.904–1.027	0.252
BMI (kg/m^2^)	1.078	0.884–1.314	0.460
Vitamin D supplementation (weekly dose)		–	0.004
No supplementation	1		
≤7000 IU/week	27.952	3.247–240.659	0.002
>7000 IU/week	6.648	0.622–71.009	0.117
Time of institutionalization (months)	1.023	0.996–1.052	0.098

BMI: body mass index; CI: 95% confidence interval; OR: odds ratio.

## Data Availability

The original contributions presented in this study are included in this article. Further inquiries can be directed to the corresponding author.
